# An Intermediate Level of BMP Signaling Directly Specifies Cranial Neural Crest Progenitor Cells in Zebrafish

**DOI:** 10.1371/journal.pone.0027403

**Published:** 2011-11-15

**Authors:** Jennifer A. Schumacher, Megumi Hashiguchi, Vu H. Nguyen, Mary C. Mullins

**Affiliations:** Department of Cell and Developmental Biology, University of Pennsylvania School of Medicine, Philadelphia, Pennsylvania, United States of America; Texas A&M University, United States of America

## Abstract

The specification of the neural crest progenitor cell (NCPC) population in the early vertebrate embryo requires an elaborate network of signaling pathways, one of which is the Bone Morphogenetic Protein (BMP) pathway. Based on alterations in neural crest gene expression in zebrafish BMP pathway component mutants, we previously proposed a model in which the gastrula BMP morphogen gradient establishes an intermediate level of BMP activity establishing the future NCPC domain. Here, we tested this model and show that an intermediate level of BMP signaling acts directly to specify the NCPC. We quantified the effects of reducing BMP signaling on the number of neural crest cells and show that neural crest cells are significantly increased when BMP signaling is reduced and that this increase is not due to an increase in cell proliferation. In contrast, when BMP signaling is eliminated, NCPC fail to be specified. We modulated BMP signaling levels in BMP pathway mutants with expanded or no NCPCs to demonstrate that an intermediate level of BMP signaling specifies the NCPC. We further investigated the ability of Smad5 to act in a graded fashion by injecting *smad5* antisense morpholinos and show that increasing doses first expand the NCPCs and then cause a loss of NCPCs, consistent with Smad5 acting directly in neural crest progenitor specification. Using Western blot analysis, we show that P-Smad5 levels are dose-dependently reduced in *smad5* morphants, consistent with an intermediate level of BMP signaling acting through Smad5 to specify the neural crest progenitors. Finally, we performed chimeric analysis to demonstrate for the first time that BMP signal reception is required directly by NCPCs for their specification. Together these results add substantial evidence to a model in which graded BMP signaling acts as a morphogen to pattern the ectoderm, with an intermediate level acting in neural crest specification.

## Introduction

Neural crest cells are a multipotent population derived from embryonic ectoderm. During neurulation neural crest cells undergo an epithelial-to-mesenchymal transition, delaminate from the dorsal neural tube, and migrate throughout the embryo, contributing to a variety of tissues including craniofacial skeleton, pigment cells, and the peripheral nervous system. In both frog and chick, juxtaposition of explanted neural and non-neural ectoderm gives rise to neural crest cells [1]–[3]. Consistent with this neural-non-neural tissue interaction generating the neural crest, the zebrafish fate map reveals that neural crest cells are derived from lateral regions of the gastrula, where prospective neural tissue meets prospective epidermis [4]. Similarly in Xenopus, fate map analysis reveals that the prospective neural crest population lies adjacent to the dorsolateral marginal zone at an early gastrula stage [5]. Consistent with these studies, the earliest genes specifically expressed within the neural crest progenitor cells (NCPC), e.g. *snail, AP2*, and *foxd3*, are localized to lateral regions of the neural plate adjacent to the non-neural ectoderm [6]–[12].

Gain-of-function studies in chick and *Xenopus* have addressed the molecular nature of the signals that are involved in the induction of the neural crest. These studies have implicated Bone Morphogenetic Protein (BMP) signaling, among other signals such as Wnt and FGF, as necessary in this inductive process [9], [13], [14]. BMPs are postulated to pattern the ectoderm of zebrafish and *Xenopus* in a gradient fashion, such that high levels of activity induce epidermis, intermediate levels induce neural crest, and the absence of BMP activity is required for neurectoderm formation. In support of this idea, when zebrafish embryos are treated with a high concentration of dorsomorphin, a small molecule that inhibits type I BMP receptor activity, neural crest cells are absent, whereas a low concentration of dorsomorphin causes expansion of neural crest cells [15]. When *Xenopus* animal caps are excised and treated with intermediate levels of Noggin, they express the early neural crest marker *slug*, although this also requires the presence of FGF [9]. These results indicate that modest attenuation of endogenous BMP signaling can lead to neural crest induction. Other evidence for a BMP signaling gradient in the ectoderm, and evidence for an intermediate level of BMP signaling patterning lateral regions of the embryo, particularly neural crest, comes from genetic analysis in zebrafish [16]–[18]. In the strongly dorsalized *swirl/bmp2b* mutant, *foxd3, AP2*, and *snail* expression in neural crest during somitogenesis is absent, consistent with a requirement for BMP signaling in neural crest specification. In more weakly dorsalized *somitabun (sbn)/smad5* and *snailhouse^ty68a^* (*snh*)*/bmp7a* mutants, neural crest is greatly and moderately expanded, respectively, suggesting that these mutants retain an intermediate level of BMP signaling in an expanded region sufficient to specify neural crest [17]. However, the extent of the expansions has not been characterized, nor has the residual signaling in these mutants been demonstrated. Furthermore, the gradient model predicts that neural crest progenitors directly respond to the intermediate level of BMP signaling, however, this has not been addressed experimentally.

Here, we quantified the effects of reduction in BMP signaling on the number of neural crest cells by counting the number of Foxd3-positive cells in wild-type, *swirl, sbn*, and *snh^ty68a^* embryos to show that the expansion of the neural crest domain is not due to impaired morphogenesis but rather an increase in neural crest cell number. We modulate BMP signaling levels by over-expression of BMP antagonists in wild type and various mutant conditions to demonstrate that different levels of BMP signaling remain in *sbn* and *snh^ty68a^* mutants. We further investigate the ability of Smad5 to act in a graded fashion by injecting *smad5* antisense morpholinos and show that its dose-dependent loss recapitulates the BMP mutant phenotypes, consistent with Smad5 acting directly in neural crest progenitor specification. Using Western blot analysis, we show that P-Smad5 levels are reduced in *smad5* morphants in a dose-dependent manner, consistent with an intermediate level of BMP signaling acting through Smad5 to specify the neural crest progenitors. Finally, we perform chimeric analysis to show that BMP signaling is directly required within neural crest progenitor cells for their specification. Together these results add substantial evidence to a model in which graded BMP signaling acts as a morphogen to pattern the ectoderm, with an intermediate level responsible for neural crest specification.

## Results

### Reduction of BMP signaling in the *swr, sbn*, and *snh* mutants affects NCPC specification

**Figure 1:**
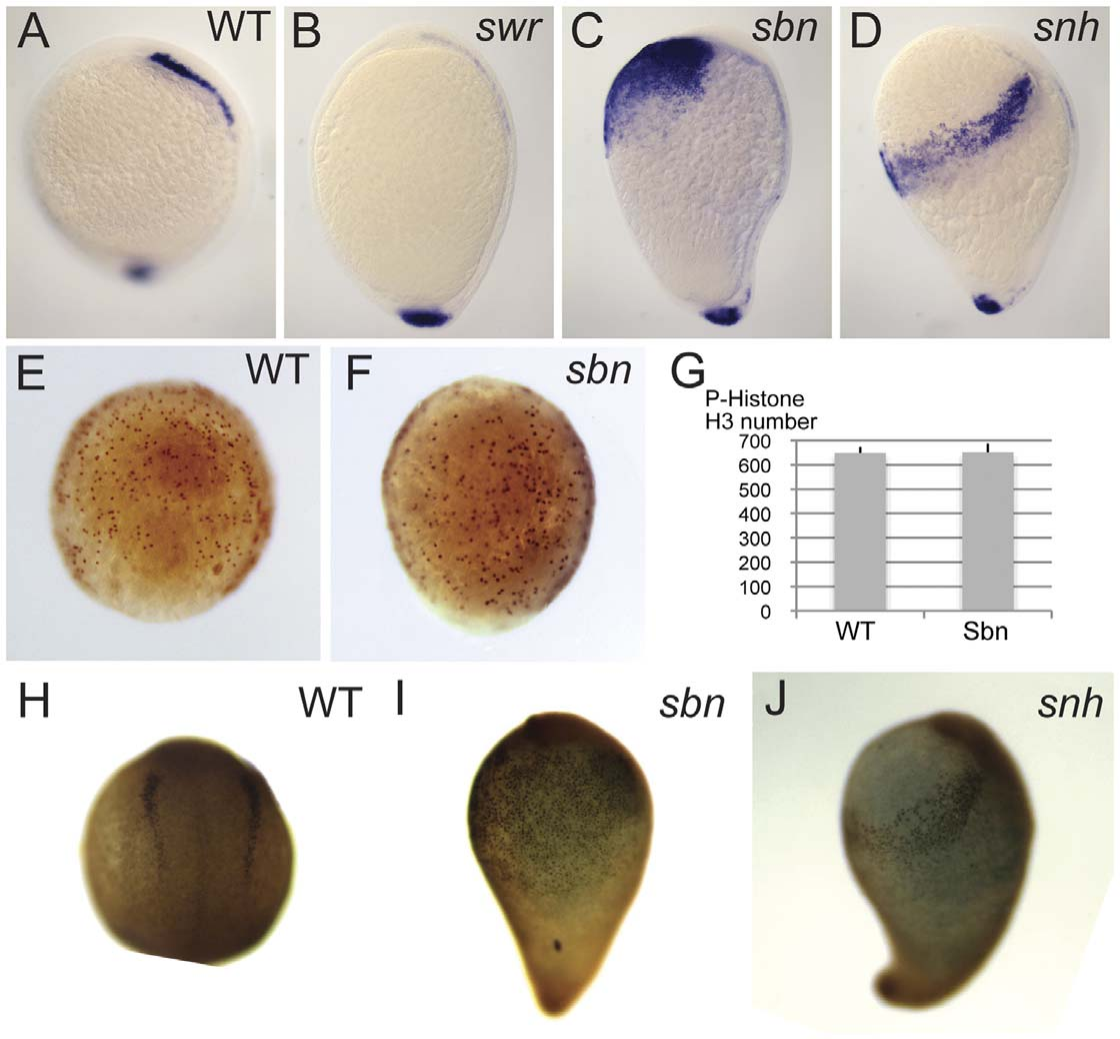
Reduction of BMP signaling in BMP pathway mutants affects the number of NCPC specified. *foxd3* expression at bud stage in wild-type (A), *swr* (B), *sbn* (C), and *snh^ty68a^* (D). P-histone H3 expression at 80% epiboly stage in wild-type (E) and *sbn* (F). (G) Quantification of the number of P-histone H3 positive cells in wild-type (n = 3) and *sbn* (n = 3). Foxd3 protein expression at the 2-somite stage in wild-type (H), *sbn* (I), and *snh^ty68a^* (J).

We reported previously that the NCPC domain is decreased in *swr*/*bmp2b*, and increased in *sbn^dtc24^*/*smad5* and *snh^ty68a^*/*bmp7a* mutant embryos [17]. This analysis was done during early somitogenesis stages, several hours after *foxd3*, the earliest neural crest marker, is expressed. To investigate if these defects are due to defects in NCPC specification, we examined *foxd3* expression at the end of gastrulation (bud stage) in these BMP pathway mutants. We found that NCPCs were greatly reduced to absent in *swr*/*bmp2b* mutants (Fig. 1B); greatly expanded in *sbn*/*smad5* (Fig. 1C); and moderately expanded in *snh^ty68a^*/*bmp7a* mutant embryos (Fig. 1D) compared to wild-type (Fig. 1A). These results are consistent with the hypothesis that mutations in BMP pathway components affect specification, rather than maintenance of neural crest.

BMP signaling has been implicated in both promoting and inhibiting cell proliferation (reviewed in [19]–[22]). To determine if the expanded number of NCPC observed in *sbn* mutants reflects an increase in their proliferation, we examined the expression of Phospho-Histone H3, a marker of proliferating cells, in wild-type and *sbn/smad5* mutant embryos. By gross inspection, we did not detect a difference between the mutants and wild type at any point throughout gastrulation (50%, 70% and 90% epiboly stages, and bud stage, data not shown). We counted the number of Phospho-Histone H3 positive cells at mid-gastrulation (70-80% epiboly) in wild-type (n = 3, Fig. 1E) and *sbn/smad5* mutants (n = 3, Fig. 1F) and found no correlation between expanded NCPCs and an increase in proliferation (Fig. 1G). Hence, an increase solely in cell proliferation cannot account for the large increase in NCPCs in *sbn/smad5* mutants. Rather these results are consistent with an enlarged domain of cells that is specified as NCPC in *sbn/smad5* mutants.

### The number of NCPC is increased in *sbn/smad5* and *snh/bmp7a* mutants

In 5-somite stage wild-type embryos, the anterior neural crest population is 2–3 cell layers thick, and thins to a single cell layer in the posterior (data not shown). It has previously been shown that dorsal convergence is impaired in several BMP pathway mutants [23], [24], thus the apparent increase in the neural crest population in these mutants could be due in part or entirely to a failure of the NCPCs to converge into a multilayer tissue rather than an actual increase in cell number. To determine the number of NCPC in *sbn/smad5* and *snh^ty68a^/bmp7a* mutants compared to wild-type, we counted the number of Foxd3-positive NCPCs (Fig. 1H–J). Foxd3 protein localizes to the nucleus, allowing one to easily count individual cells, particularly in regions with multiple cell layers [25]. Foxd3 protein detection is delayed compared to *foxd3* RNA. Thus, we counted Foxd3-positive nuclei in 2-somite stage embryos, the earliest time point exhibiting strong Foxd3 expression (Fig. 1H). The average number of neural crest cells in wild-type was 403 (n = 4), and in *snh^ty68a^* was 1160 (n = 4), or a 2.8-fold increase over wild-type. *sbn/smad5* embryos averaged 1965 neural crest cells (n = 4), or a 4.8-fold increase over wild-type. Thus, the larger apparent neural crest domain in *snh^ty68a^/bmp7a* and *sbn/smad5* mutant embryos reflects a larger number of NCPCs. The NCPCs in these mutants also occupy a larger area than expected based on their increased number due to reduced dorsal convergence of the NCPC in these mutants, i.e. most of the NCPC were found in a single rather than a multi-cell layer. Together, these results support a model in which an intermediate level of BMP signaling is present in a larger domain in these mutants than in wild-type embryos.

### Reducing BMP signaling in wild-type embryos dose-dependently affects NCPC phenotypes

**Figure 2:**
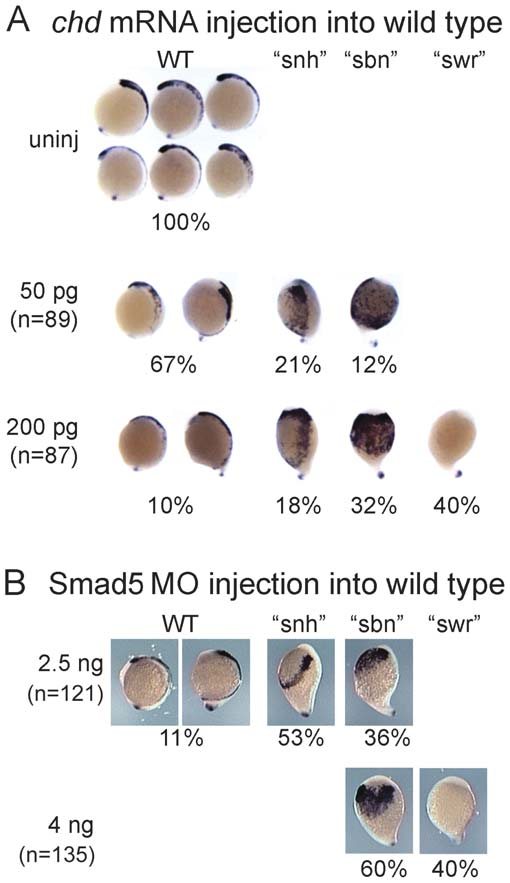
Reduction of BMP signaling in wild-type embryos causes expansion or loss of NCPC in dosage-sensitive manner. *foxd3* expression in *chd* mRNA injected embryos (A) and *smad5* MO injected embryos (B) at the end of gastrulation. (A) Injection of a low dose of *chordin* mRNA (50 pg) generates weaker NCPC phenotypes (WT = normal or very mild expansion, “*snh*”  =  moderate expansion), whereas a high dose (200 pg) leads to strong phenotypes (“*sbn*” = large expansion, or “*swr*”  = loss). (B) Injection of a low 2.5 ng dose of *smad5* MO leads to “*snh*” and “*sbn*” phenotypes. Injection of a high 4 ng dose of *smad5* MO leads to “*sbn*” and “*swr*” phenotypes exclusively.

Based on our previously proposed model, we predict that moderate or strong inhibition of BMP signaling in wild-type embryos will lead to an expansion or loss of NCPC, respectively. To test this prediction, we decreased BMP signaling in wild type embryos by injecting mRNA encoding either a truncated *Xenopus* BMP receptor (*tBR*, [26]) or zebrafish *chordin* (Fig. 2A, [27]), an extracellular BMP antagonist [28]. Both of these methods yielded similar results. As expected, over-expression of either inhibitor produced a range of dorsalized phenotypes. We classified weakly dorsalized embryos that displayed a roughly normal NCPC phenotype as “wild-type”. Upon injection of 50 pg of *chordin* mRNA into wild-type embryos (n = 89), we observed that 67% of the embryos exhibited a wild-type (WT) NCPC phenotype, 21% the “*snh*” and 12% the “*sbn*” phenotype (Fig. 2A, see Fig 1A–D for the classification of “wild-type”, “*snh*”, “*sbn*”, and “*swr*” phenotypes). When we increased the amount of *chordin* mRNA injected to 200 pg (n = 87), 40% of the embryos displayed the “*swr*” phenotype; 32% the “*sbn*”; 18% the “*snh*” phenotype; and only 10% exhibited a wild-type NCPC phenotype.

The *sbn^dtc24^* mutation used in these neural crest studies is an antimorphic allele of *smad5*, raising the possibility that the large expansion of neural crest in these embryos is due to dominant-negative effects interfering with Smad proteins that are used by other signaling pathways acting in NCPC specification. To confirm that the *sbn^dtc24^* phenotype is due to a reduction in BMP-responsive Smad5 activity only, and to determine if Smad5 itself can act in a dose-dependent fashion, we injected various amounts of a previously described translation-blocking *smad5* antisense morpholino oligonucleotide into wild-type embryos [29]. Injection of 2.5 ng of *smad5* MO1 led to embryos with a range of moderately to largely expanded neural crest populations, whereas 4 ng of *smad5* MO1 led to a large expansion or loss of neural crest cells (Fig. 2B). Taken together, these results indicate that reducing BMP signaling dose-dependently recapitulates the NCPC phenotypes observed in the BMP mutants. Furthermore, the results indicate that the gradient of BMP signaling that specifies NCPCs acts primarily through Smad5, and that Smad5 itself can act in a graded fashion to specify NCPCs.

### The NCPC phenotypes of *swr/bmp2b, sbn/smad5*, and *snh/bmp7a* mutants correlate with distinct residual BMP signaling levels

**Figure 3:**
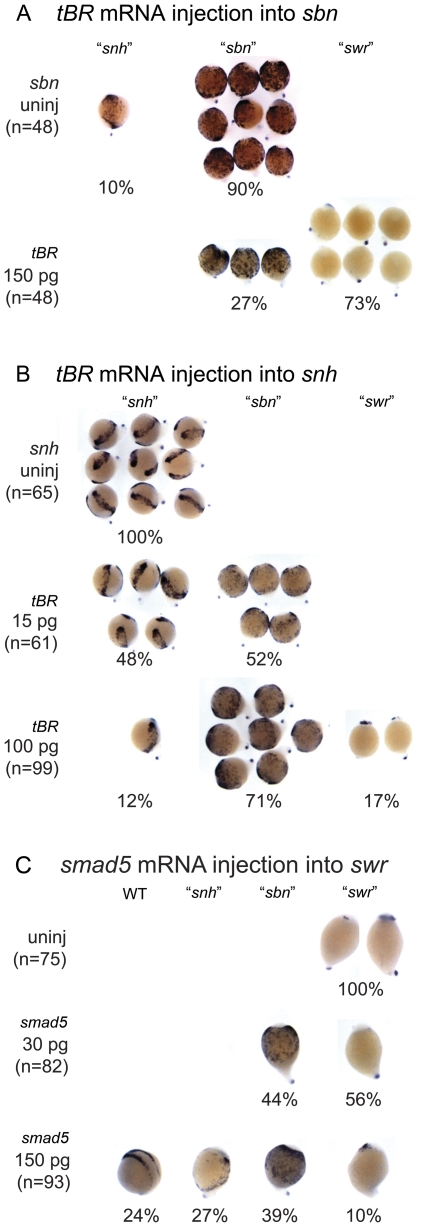
NCPC domains when BMP signaling is reduced in *somitabun* and *snailhouse* and increased in *swirl* embryos. *foxd3* expression at the end of gastrulation in *tBR* mRNA injected embryos of *sbn* (A) and of *snh* (B), and *smad5* mRNA injected into *swr* embryos (C). (A) Injection of *tBR* mRNA into *sbn* mutants leads to the majority of embryos displaying a “*swr*” phenotype. (B) Injection of a low 15 pg dose of *tBR* mRNA into *snh* mutants leads to nearly equal numbers of “*snh*” and “*sbn*” phenotypes. Injection of a higher 100 pg dose leads to the majority of embryos displaying the “*sbn*” phenotype and also a percentage displaying a stronger “*swr*” phenotype. (C) Injection of a low 30 pg dose of murine *smad5* mRNA results in nearly half of embryos displaying a “*sbn*” phenotype. Injection of a higher 150 pg dose results in a small percentage of embryos displaying the “*swr*” phenotype, and the rest of the embryos divided between “*sbn*”, “*snh*”, and WT phenotypes.

We previously proposed a model in which an intermediate level of BMP signaling specifies NCPCs, and that BMP signaling is lowered below this level in *swr/bmp2b* mutants, resulting in a loss of NCPCs. Furthermore, we predict that different intermediate amounts of residual BMP signaling are present in *sbn/smad5* and *snh/bmp7a* mutants, leading to the great and moderate expansion of NCPCs, respectively, in these mutants [17]. To test our hypothesis that the NCPC phenotypes of *swr/bmp2b*, *sbn/smad5*, and *snh/bmp7a* reflect the relative amounts of BMP signaling in these mutants, we decreased or increased the amount of BMP signaling in these mutants and examined the effects on NCPCs. Having established that *tBR* and *chordin* over-expression can recapitulate the BMP mutant phenotypes in a dose-sensitive fashion, we used this technique to further decrease BMP signaling in the BMP pathway component mutants. We predicted that decreasing BMP signaling in *sbn/smad5* or *snh^ty68a^/bmp7a* mutants would phenocopy the NCPC phenotype of *swr* mutants or *sbn and swr* mutants, respectively, in a dose-dependent manner. Fig. 3A shows a representative experiment in which we injected *tBR* into embryos from a cross between two *sbn/smad5* heterozygous fish. Since *sbn/smad5* is a fully penetrant dominant maternal-effect mutation [30], all offspring from this cross exhibit the mutant phenotype. In the uninjected group (n = 48), 90% of the embryos exhibit a “*sbn*” NCPC phenotype in which there is a great expansion of NCPC and 10% display a “*snh*” NCPC phenotype, a moderate expansion of NCPC. However, injection of 150 pg of *tBR* into these mutants (n = 86) resulted in a reduction in the number of NCPCs. Only 27% of embryos exhibited the “*sbn*” NCPC phenotype, whereas the majority (73%) showed a great reduction of NCPCs, resembling *swr* mutants.

It is possible that the loss of *foxd3* expression reflects a general deleterious effect on development and not a stronger dorsalization of *sbn/smad5* mutant embryos. To address whether over-expression of *tBR* caused a general reduction in gene expression, we examined the expression of *krox20*, a marker of prospective hindbrain rhombomeres 3 and 5 [31]. We found that *sbn/smad5* mutant embryos injected with the *tBR* mRNA expressed *krox20* and, in fact, both rhombomeres were greatly expanded in these embryos in a manner similar to that of *swr/bmp2b* mutants (data not shown, [17]). From these results, we conclude that there is residual BMP signaling in *sbn/smad5* embryos, thus reducing BMP signaling in these embryos phenocopies the “*swr*” NCPC phenotype.

We next reduced the level of BMP signaling in *snh/bmp7a* mutant embryos to ask whether this would result in the NCPC phenotype observed in *sbn/smad5* or *swr/bmp2b* mutants. We injected embryos from a cross between two rescued homozygous *snh/bmp7a* adult fish; thus all progeny display a mutant phenotype. As expected, the uninjected embryos exhibited a moderate expansion of NCPC typical of the “*snh*” phenotype (Fig. 3B). When we injected a low amount (15 pg) of *tBR* mRNA (n = 61), 52% of the embryos showed the “*sbn/smad5*” NCPC phenotype; whereas only 48% exhibited the “*snh*” NCPC phenotype (Fig. 2B). When we injected a high amount (100 pg) of tBR mRNA, the strength of the NCPC phenotype increased: 17% of the *snh* homozygous embryos exhibited the “*swr*” NCPC phenotype; 71% displayed the “*sbn*” phenotype; and only 12% showed the “*snh*” phenotype seen in the uninjected siblings. Thus, we find that lowering BMP signaling in *snh^ty68a^* mutant embryos can phenocopy the great expansion of the NCPCs observed in *sbn/smad5* mutants, and that a further reduction results in loss of NCPCs in a small percentage of embryos. We note here that with all of these experiments, we consistently found embryos that appeared intermediate between either the "*swr*" and “*sbn*" or the "*sbn*" and "*snh* " NCPC phenotypes. In these cases, we classified the embryos into the group they most closely resembled.

We also increased BMP signaling in a dose-dependent manner in the *swr/bmp2b* mutant by over-expressing *smad5* mRNA to determine if this could produce the NCPC phenotype of *sbn/smad5* and *snh^ty68a^/bmp7a* mutants. Following injection of 30 pg of murine *smad5* mRNA (n = 82), we found that 44% of the embryos displayed the expanded NCPC phenotype of “*sbn*” mutants, and the remaining mutants appeared unchanged. When we increased the amount to 150 pg (n = 93), we observed a range of phenotypes from the "*swr*" to wild-type NCPC phenotypes (Fig. 3C). Therefore, we conclude that increasing BMP signaling in *swr/bmp2b* mutants leads to an expansion of NCPC in a dosage-sensitive manner.

### Phosphorylated Smad1/5 levels correlate with the strength of NCPC phenotype in *smad5* morphant embryos

To further examine the extent of reduction in BMP signaling in *smad5* morphant embryos, we examined phosphorylated Smad1/5 (P-Smad1/5) levels. We injected *smad5* translation-blocking morpholinos (MOs) into wild-type embryos at different concentrations and examined P-Smad1/5 levels by Western blot analysis at the 60% epiboly stage, just after cranial NCPC are specified [32]. For each injection dose, a fraction of the embryos were used for Western blot analysis and the remaining embryos were examined for the NCPC phenotype.

**Figure 4:**
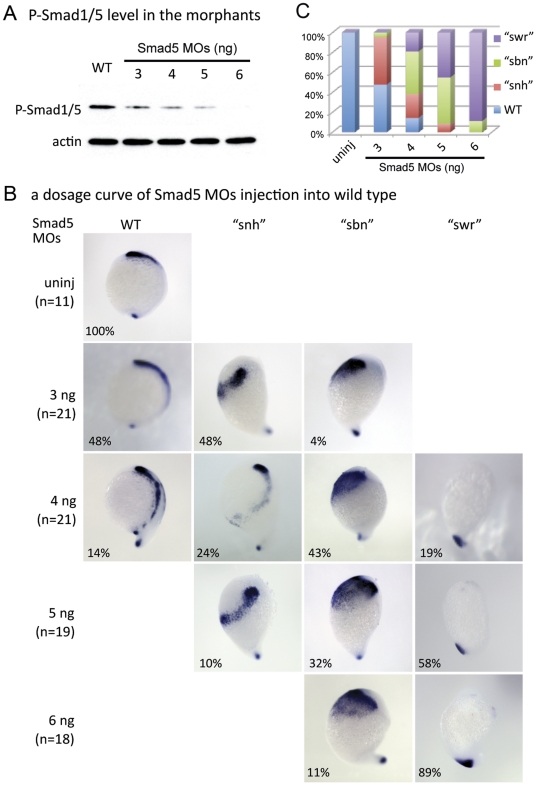
Levels of P-Smad1/5 correlate with the strength of NCPC phenotype in *smad5* morphant embryos. (A) P-Smad1/5 levels in *smad5* morphant embryos. Embryos injected with increasing higher concentrations of *smad5* MOs show decreasing P-Smad1/5 levels relative to uninjected controls. Actin was used as a loading control. (B, C) Expression of *foxd3* in embryos injected with increasing doses of *smad5* MOs. Injection of a low dose of *smad5* MOs (3 ng) leads to WT, “*snh*” and “*sbn*” phenotypes, whereas injection of higher doses of *smad5* MOs (5 ng, 6 ng) lead to “*sbn*” and “*swr*” phenotypes. The embryos used for in situ hybridization of *foxd3* in Fig. 4B are from the same batch of injected embryos used for Western blotting in Fig. 4A.

Compared with wild-type embryos, all *smad5* morphant embryos displayed lower P-Smad5 levels that correlated with the *smad5* MO dose injected. Embryos injected with 6ng of *smad5* MOs had no detectable P-Smad1/5 compared to embryos injected with 5 ng, 4 ng and 3 ng of *smad5* MOs, which had progressively increasing levels of P-Smad1/5 (Fig. 4A).

We examined expression of *foxd3*, one of the earliest markers of NCPC, at the end of gastrulation in the remaining embryos of each group to determine the extent of expansion of, or loss of NCPC at each *smad5* MO dose. This allowed us to correlate the NCPC phenotype with P-Smad1/5 levels during gastrulation. Injection of a low dose of *smad5* MOs (3 ng) caused primarily WT and “*snh*” phenotypes, whereas higher doses of *smad5* MOs (5 ng, 6 ng) resulted in “*sbn*” and “*swr*” phenotypes almost exclusively (Fig. 4B, 4C). We find that increasing amounts of *smad5* MOs leads to decreasing P-Smad1/5 levels, which correlates with expansion of NCPCs at intermediate P-Smad1/5 levels and then a loss of NCPCs at undetectable P-Smad1/5 levels, consistent with the BMP mutant results.

### BMP signaling is autonomously required for neural crest progenitor cell specification

Models of BMP gradient activity predict that each cell type within the gradient field responds *directly* to the level of BMP signaling, thus neural crest progenitor cells are expected to respond directly to an intermediate level of BMP signaling. The direct response of NCPCs to BMP signaling has not been addressed in any organism. We addressed the cell autonomy of BMP signaling in NCPCs by placing donor cells that cannot respond to a BMP signal into a wild-type host environment where BMP signaling is intact and determined if the donor cells can express Foxd3. If the donor cells can express Foxd3, this indicates that BMP signaling is not acting directly to specify NCPCs. Alternatively, if donor cells do not express Foxd3 this would indicate that BMP signaling directly induces NCPCs.

**Figure 5:**
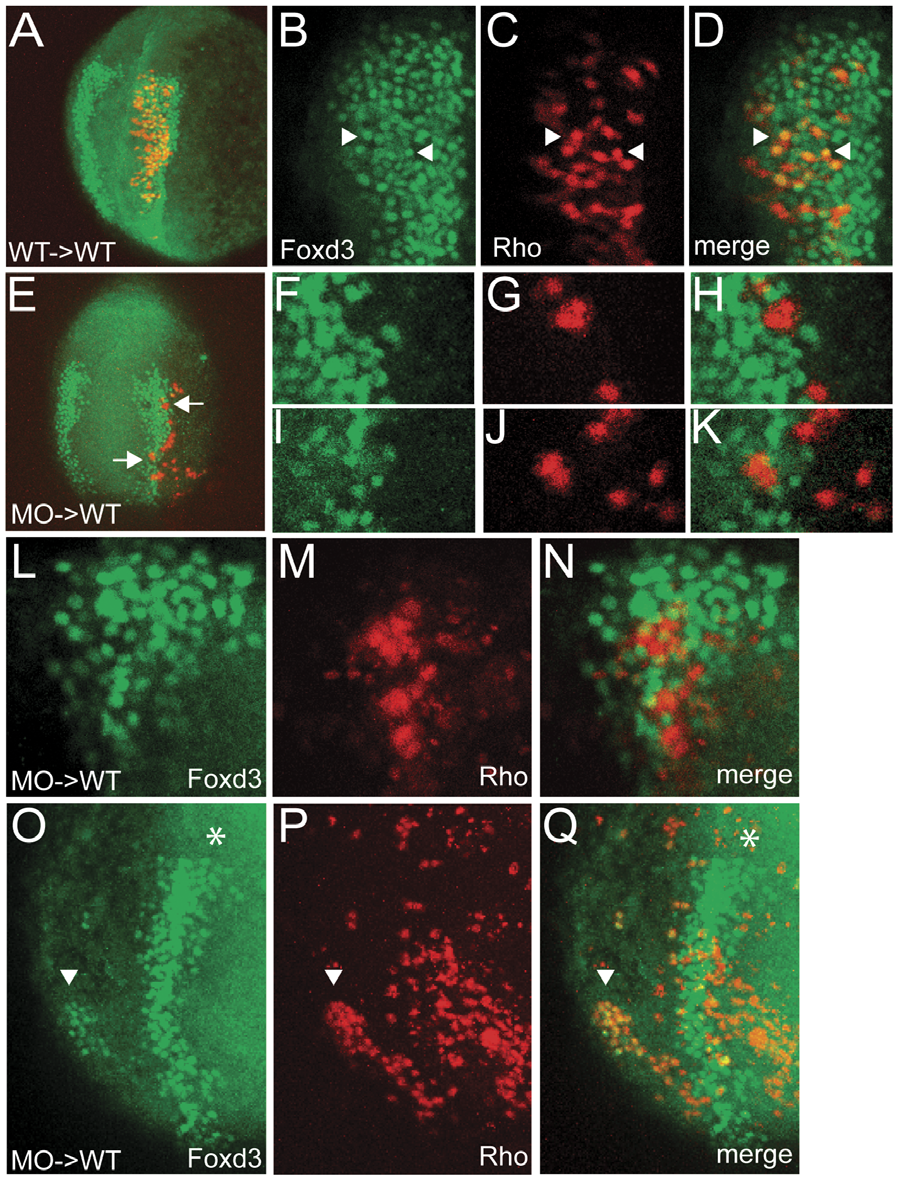
BMP signaling is required cell autonomously in NCPCs for their specification. (A) Z-projection of confocal sections showing that wild-type donor cells transplanted into wild-type hosts readily express the neural crest marker Foxd3 at the 3-somite stage. (B,C,D) Single confocal section of the embryo in A. Foxd3 (B) and lineage tracer rhodamine dextran (C) are found in the same cells (D, arrowheads). (E) Z-projection of confocal sections showing that *smad5* morphant donor cells within the neural crest region of a wild-type host do not express the neural crest marker Foxd3. (F, G, H, I, J, K) Single confocal sections of the cells indicated by arrows in E. Foxd3 (F, I) and lineage tracer (G, J) do not colocalize (H, K). (L, M, N) Single confocal section of a different host embryo containing *smad5* morphant cells (Rho) within the neural crest region that do not express Foxd3. (O, P, Q) Z-projection of confocal sections of chimera in which donor cells were induced as ectopic neural crest. Foxd3 (O) and lineage tracer (P) colocalize (Q) in a patch of cells (arrowhead) located ventrally from the normal neural crest domain (asterisk).

To generate donor cells that cannot respond to BMP signaling, we used a combination of *smad5* morpholinos to inhibit Smad5 translation. We then transplanted 5–15 cells from blastula-stage donors to a region above the margin of a blastula-stage wild type host embryo, in a region predicted to become neural crest based on fate-mapping studies [4]. Donor embryos were analyzed individually for *foxd3* mRNA expression, and only chimeras derived from donors that completely lacked *foxd3* expression were further analyzed. As a control, we transplanted wild-type cells into the same region of blastula-stage wild-type host embryos, and examined Foxd3 expression at the 3-somite stage. In 7 of 7 chimeras analyzed, these wild-type donor cells readily expressed Foxd3 (Fig. 5A–D). In 8 of 9 embryos with *smad5* morphant clones in the neural crest area, donor cells did not express Foxd3. Clones in two separate embryos from two different donors are shown in Figure 5E–N. In the single embryo in which donor cells expressed Foxd3, donor cells became neural crest cells only ectopically in a more ventral region to the normal domain (Fig. 5O–Q). Ectopic neural crest induction was never observed with wild-type donor cells. It is possible that the morphant-derived cells retained a slight amount of Smad5, insufficient in the donor to specify neural crest but sufficient to weakly respond to a surrounding high level of BMP signaling ventrally in the chimera; responding at the lower level appropriate for neural crest rather than epidermis, which would normally occupy the domain. Taken together, these results indicate that NCPCs directly respond to BMP signaling.

## Discussion

### A low level of BMP signaling is required for specification of neural crest progenitors

Temporal inhibition of BMP signaling using Tg(*hsp70:chd*) [32] and treatment of embryos with dorsomorphin at different time points [15] show that BMP signaling acts in cranial neural crest specification at an early gastrula stage. Cells become committed in the zebrafish embryo between the shield stage and 80% epiboly [33], [34]. NCPC phenotypes are evident in BMP pathway mutants at bud stage, shortly after cell commitment, suggesting that BMP signaling is involved in the primary specification of these cells rather than maintenance of this cell type.

We previously reported that there are no differences in cell death between wild-type and BMP pathway mutants during blastula and gastrula stages [17]. Thus, cell death likely does not contribute to reduction of NCPCs in *swr/bmp2b* mutants. Our phospho-histone H3 data revealed no correlation between the increase in number of NCPCs *in sbn/smad5* mutants and cell proliferation. Taken together, these results indicate that cell proliferation and cell death do not contribute to the expansion or loss of NCPCs, and that these phenotypes reflect the specification of NCPCs based on the level of BMP activity in each mutant.

**Figure 6:**
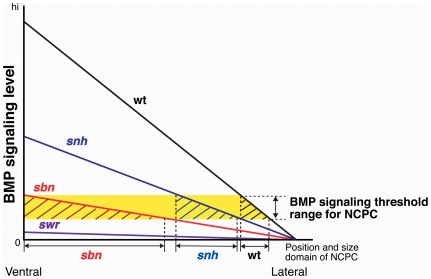
BMP gradient model for NCPC specification. The Y axis shows BMP signaling levels. The X axis indicates position along the dorsoventral axis. The threshold range of BMP signaling that specifies NCPC is shown in yellow. The intersection of the gradient with the threshold range for NCPC specification leads to NCPC formation in a lateral region in the size domain shown. In WT, the gradient of BMP signaling reaches a high level ventrally and NCPCs are located in a lateral region of the embryo where BMP signaling levels are low. The region of NCPCs specified in WT is shown with black stripes over the yellow area. In *snh*, the BMP signaling gradient is lower than WT. Therefore, the NCPCs in *snh* (blue striped area) are slightly expanded compared to wild type and are located in a more ventral region than WT. In *sbn*, the BMP signaling gradient is lower than *snh*. The NCPCs are located in a more ventral region than *snh* and the NCPCs in *sbn* (red striped area) are greatly expanded compared to wild type. In the *swirl/bmp2b* mutant, BMP signaling level is absent or very low, leading to the great reduction or absence of NCPCs.

Based on our results, we propose a model to explain the NCPC phenotypes displayed by BMP pathway mutants, shown in Figure 6. Our results indicate that a low level of BMP signaling specifies NCPC. This level would be found throughout much of *sbn/smad5* mutant embryos, thereby directing ectodermal cells located in ventral and lateral regions of this mutant towards a NCPC fate. Our data suggest that the level of BMP signaling in *swr/bmp2b* mutants is lower than in *sbn/smad5* mutants, very likely near or at the threshold of BMP activity required for NCPC specification, since weak *swr/bmp2b* mutants retain a few NCPCs. In the case of *snh^ty68a^/bmp7a* mutant embryos, our results indicate that the level of BMP signaling is highest among the three mutants, but lower than in wild-type embryos. If a low level of BMP signaling specifies the NCPCs, then our model would predict a moderate expansion of NCPCs in *snh^ty68a^/bmp7a* mutant embryos, consistent with their phenotype.

### Integrating our model with other studies of NCPC specification

Experiments in amphibians and chick in which neural and epidermal tissues are juxtaposed either *in vivo* or *in vitro* led to a model in which neural crest is induced at the border between neural and non-neural ectoderm [2], [3]. In these experiments, neural tissue is a source of the secreted BMP antagontists Chordin, Noggin, and Follistatin. It is likely that these antagonists diffuse across the border into epidermal tissue and inhibit the BMP ligands there to establish an intermediate level of BMP activity at the border, which would then induce neural crest formation. This scenario mimics the proposed mechanism for formation of the BMP activity gradient in vivo, which generates a low level of BMP signaling in lateral regions of the gastrulating embryo, precisely where NCPC are located. Thus, our results are consistent with the previously proposed model. Importantly, our chimeric analysis extends this model to show that neural crest progenitor cells require an intact BMP signaling pathway cell autonomously, revealing for the first time that NCPCs directly respond to BMP signaling.

In zebrafish Wnt signal reception is also required autonomously within neural crest cells, and loss of *wnt8* results in a loss of neural crest [25]. It is possible that an intermediate level of BMP signaling makes prospective neural crest cells competent to receive a second inducing signal, perhaps a Wnt signal. In support of this idea, conjugation of *Xenopus* wild type animal caps to animal caps expressing *chordin* weakly induces neural crest markers *snail* and *Xslug*, whereas conjugation of animal caps expressing *chordin* to those expressing *wnt8* strongly induces *Xslug* expression [14]. In Xenopus embryos, *Gbx2*, the earliest factor in neural crest induction, is a direct target of Wnt signaling [35]. Furthermore, during gastrulation in zebrafish, *wnt8* expression extends from the margin towards the animal pole in lateral regions of the embryo [25], where BMP signaling is predicted to be present at intermediate levels, thus making it a good candidate for the second inducer.

Wnt signaling is also a posteriorizing factor, and along with FGF and retinoic acid, may specify neural crest in the BMP-induced competent lateral regions by nature of their posteriorizing activity. Consistent with this, anterior lateral ectoderm, or neural fold, which normally becomes forebrain, not neural crest, can be induced to express neural crest markers by treatment with Wnt, FGF, or retinoic acid (RA) [36]. Knockdown of FGF and RA signaling strongly reduce expression of *midkine-b*, which regulates cell specification at the neural plate border, whereas activation of Wnt signaling enhances the expression [37]. However, β-catenin can also expand neural crest in whole embryos without posteriorizing neural tissue [38]. Our results are consistent with either of these models.

### The interpretation of graded BMP signaling

Our observation that BMP signaling is required cell autonomously by neural crest cells highlights the interesting question of how a subset of cells within a gradient field can interpret a specific level of signal to activate the appropriate downstream targets. The mechanism for BMP gradient interpretation is poorly understood in vertebrates. Our result that reduction of P-Smad1/5 levels leads to expansion and loss of neural crest in a concentration-dependent manner is consistent with a scenario in which differential activation of downstream targets at least in part leads to a distinct domain of neural crest gene expression. However, *foxd3* is not known to be a direct target of the BMP pathway, thus there are likely other downstream targets through which an intermediate level of BMP signaling leads to *foxd3* expression in neural crest.

We previously proposed that the gastrula BMP gradient generates a pattern of nested gene expression in ventral and lateral regions of the embryo, which in turn would reciprocally regulate each other to generate more restricted domains of expression by the end of gastrulation. In this model *AP2* expression becomes restricted to lateral regions of the neural plate by the end of gastrulation [17]. It is possible that *AP2* provides a link between BMP signaling and neural crest induction. In support of this notion, ectopic expression or morpholino knockdown of *AP2* leads to induction or reduction, respectively, of the neural crest specific genes Slug and Sox9 in *Xenopus* [8]. Furthermore, simultaneous loss of two AP2 genes in zebrafish, *tfap2a* and *tfap2c*, reveals a cell autonomous requirement for AP2 in NCPC specification [39], [40]. Another potential link between BMP signaling and neural crest gene expression is the Msx family, which has been shown to be a direct target of BMP signaling [41], [42]. *msxB* expression is affected in BMP mutants in a similar, yet even more sensitive manner than *foxd3* [43].

The results we present here provide evidence that an intermediate level of BMP signaling specifies neural crest progenitor cells. We also present the first evidence that BMP signaling directly specifies neural crest progenitor cells. These results will advance the further study of the action of BMP signaling in neural crest specification and its interaction with other pathways regulating neural crest specification.

## Materials and Methods

### Ethics Statement

All of the zebrafish studies were performed in accordance with, and with approval from, the Institutional Animal Care and Use Committee of the Office of Regulatory Affairs at the University of Pennsylvania.

### Fish strains and breeding

Incrosses were performed between heterozygous fish of the following mutants: *swr^tc300a^*, *swr^ta72^*, *swr^tdc24^*, *snh^ty68a^*, and *sbn^dtc24^* [30], [44]. Embryos were also obtained from incrosses between rescued homozygous *swr^tc300a^*, *swr^ta72^*, and *snh^ty68a^* adults. *swr (bmp2b)* and *snh (bmp7a)* mutant embryos were determined at bud stage or early somitogenesis stages by their elongated, American football-shaped morphology. All progeny of *sbn (smad5)* heterozygous females are mutant, due to the dominant maternal-effect nature of this mutation [30].

### In situ hybridization, immunohistochemistry, cell counting

Whole-mount in situ hybridization was performed as previously described using the *foxd3* probe (previously called *fkd6*, [10]). Foxd3 protein was detected using a 1∶1000 dilution of Foxd3 rabbit antisera in 10% NCS-PBST (10% fetal bovine serum, 1% DMSO, 0.1% Tween 20 in PBS) as described [25]. Following several washes in PBST, embryos were developed using diaminobenzidine according to the manufacturer's directions (Vector Labs). For Foxd3 cell counting experiments, stained embryos were fixed in 4% paraformaldehyde-PBST for two days at room temperature, then cleared in benzylbenzoate∶benzylalcohol (2∶1), de-yolked using watchmaker forceps, and flattened onto glass slides in Canada Balsam. Embryos were viewed using Nomarski optics on a Leica Axioskop. Phospho (P)-histone H3 staining was performed as previously described [45]. For P-histone H3 cell counting experiments, stained embryos were cleared in 100% glycerol. To count the number of P-histone H3 positive cells in the whole embryo without duplication, several nuclei were first selected as landmarks in the embryo and images taken. Then the embryo was rotated slightly and several new nuclei were selected for landmarks and images taken. Rotation of the embryo, selection of landmarks and image acquisition were repeated until returning to the first landmark. Images were captured using a Kontron digital camera and processed using Adobe software using the nuclei landmarks to overlap images. Cells were counted from printed images.

### mRNA and morpholino injections

mRNA encoding *tBR* [26], *chordin* [27], *mSmad5* [46], and zebrafish *smad5* was synthesized using the SP6 mMessage mMachine Kit (Ambion). mRNA injections were directed into the yolk of 1–4 cell stage embryos, and were each performed at least three times, all with results similar to experiments shown. *smad5* morpholino antisense oligonucleotides (Gene Tools) were injected into the cell of 1-cell stage embryos from a cross of *sbn^m169^* heterozygotes for transplant experiments and into wild-type embryos for all other experiments. Concentration of MOs in Fig. 4 shows total combined concentration of equal amounts of *smad5* MO1 and *smad5* MO3. The concentrations are different from Fig. 2B, due to different *smad5* morpholino production lots used. *smad5* MO1 sequence is ATGGAGGTCATAGTGCTGGGCTGC, *smad5* MO3 sequence is GCAGTGTGCCAGGAAGATGATTATG.

### Western Blot Analysis

Phospho-Smad1/5 Western blots were performed as previously described [32], [47], except that the primary antibody was applied for one overnight period at a 1∶500 dilution. After the P-Smad1/5 Western, P-Smad1/5 antibody was stripped from the membrane and Actin antibody was re-probed, as a control.

### Transplantation Analysis

Donor embryos were injected with a combination of *smad5* MO1 (3 ng) and *smad5* MO3 (2–3 ng), as well as a 2.5% solution of lysine-fixable rhodamine-dextran and 2.5% solution of biotinylated dextran. Approximately 5–15 cells at the blastula stage were transplanted into the marginal region of blastula-stage, unlabelled hosts. Donor embryos were fixed at the bud stage and analyzed for the presence of neural crest by *foxd3* in situ hybridization. Host embryos were fixed at the 3–4 somite stage. Foxd3 was detected using a 1∶500 dilution of Foxd3 rabbit antisera [25] in 20% NCS-PBST followed by a 1∶500 dilution of anti-rabbit Alexa 488 in 10% NCS-PBST. Donor cells were visualized by rhodamine fluorescence. Embryos were mounted in Vectashield and imaged using a Zeiss LSM 510 confocal microscope. Images were processed using ImageJ and Adobe software.
